# *αTubulin 67C* and *Ncd* Are Essential for Establishing a Cortical Microtubular Network and Formation of the *Bicoid* mRNA Gradient in *Drosophila*

**DOI:** 10.1371/journal.pone.0112053

**Published:** 2014-11-12

**Authors:** Khalid Fahmy, Mira Akber, Xiaoli Cai, Aabid Koul, Awais Hayder, Stefan Baumgartner

**Affiliations:** Department of Experimental Medical Sciences, Lund University, Lund, Sweden; Technische Universität Dresden, Germany

## Abstract

The Bicoid (Bcd) protein gradient in *Drosophila* serves as a paradigm for gradient formation in textbooks. To explain the generation of the gradient, the ARTS model, which is based on the observation of a *bcd* mRNA gradient, proposes that the *bcd* mRNA, localized at the anterior pole at fertilization, migrates along microtubules (MTs) at the cortex to the posterior to form a *bcd* mRNA gradient which is translated to form a protein gradient. To fulfil the criteria of the ARTS model, an early cortical MT network is thus a prerequisite. We report hitherto undiscovered MT activities in the early embryo important for *bcd* mRNA transport: (i) an early and omnidirectional MT network exclusively at the anterior cortex of early nuclear cycle embryos showing activity during metaphase and anaphase only, (ii) long MTs up to 50 µm extending into the yolk at blastoderm stage to enable basal-apical transport. The cortical MT network is not anchored to the actin cytoskeleton. The posterior transport of the mRNA via the cortical MT network critically depends on maternally-expressed αTubulin67C and the minus-end motor Ncd. In either mutant, cortical transport of the *bcd* mRNA does not take place and the mRNA migrates along another yet undisclosed interior MT network, instead. Our data strongly corroborate the ARTS model and explain the occurrence of the *bcd* mRNA gradient.

## Introduction

The Bicoid (Bcd) protein is a paradigm for morphogen gradient formation taught in textbooks and studied for more than two decades. The hallmark of the Bcd morphogen is its spectacular gradient along the anterior-posterior axis of the early *Drosophila* blastoderm egg [1]. In the past, two models were put forward to explain the formation of the gradient, the ARTS model [2]–[4] and the SDD model [1], [5]. The SDD (**S**ynthesis, **D**iffusion and **D**egradation) model proposes that the gradient arises through translation of an anteriorly-localized *bcd* mRNA source, followed by diffusion of Bcd throughout the embryo, and uniform degradation. In contrast, the ARTS (**A**ctive **R**NA **T**ransport and **S**ynthesis) model proposes that the *bcd* mRNA is actively transported along the cortex in a posterior direction from its anterior pole to form an mRNA gradient which serves as template for the synthesis of Bcd.

Recently, the SDD model encountered some severe difficulties: the diffusion coefficient of Bcd was found to be two orders of magnitude too low to establish a steady-state Bcd gradient by the blastoderm stage [5]. Subsequently, other reports measured higher diffusion rates [6], [7], calculated to be high enough to explain the SDD diffusion model, and corroborated by a recent biophysical model analysis [8]. Unfortunately, as a major drawback, all the above analyses comprised measurements of diffusion during late nuclear cycles (nc) 10–14 and at the peripheral cytoplasm of the embryo. However, we should bear in mind that the time window from fertilization up to nc 10 is the important time interval where the SDD model predicts long-range diffusion of Bcd. Arguably, the diffusion properties of proteins in the dense yolk are different from those of the cytoplasm surrounding the cortical nuclei at 10–14 which make predictions of the diffusion coefficient problematic. It is thus important to note here that, although these above experimental and theoretical data permit to explain the SDD model, it would be premature to imply *a priori* that the SDD model is the correct one. Furthermore, the model still lacks a direct proof of the existence of long-range Bcd diffusion, e. g. by tracking single Bcd molecules during the early nuclear cycles. Equally important to note: the ARTS model does not argue against a high diffusion coefficient of Bcd, it is only largely irrelevant for the model.

The existence of a *bcd* mRNA gradient, which is the hallmark of the ARTS model, was first described in 1986 [2]. In 2009, the SDD model was challenged by a detailed analysis of the *bcd* mRNA distribution during early *Drosophila* embryogenesis [3], which confirmed previous data [2]. On the other hand, the ARTS model, which is based on this demonstration, raises the question of how the *bcd* mRNA gradient forms from a *bcd* mRNA source that at fertilization is strictly localized to the anterior pole of the embryo.

Plenty of information is meanwhile available on the transport of the *bcd* mRNA during oogenesis, using *in vivo* imaging of the movement of the *bcd* mRNA [9], [10]. These data showed that the dumping of the *bcd* mRNA by the nurse cells into the oocyte starts at stage 10b, accompanied by an active cytoplasmic movement within the oocyte (also referred to as “ooplasmic streaming” [9]. During subsequent developmental stages (stages 10b-13), the *bcd* mRNA behaves very dynamic [10], however, a microtubule (MT)-based transport, with the help of the minus-end motor *dynein*, begins to accumulate the mRNA at the anterior pole [9], [10] such that at the end of oogenesis, the *bcd* mRNA is confined completely to the anterior pole, anchored by the actin cytoskeleton. Recent measurements showed that *Drosophila* females produce around 7.4×10^5^ *bcd* mRNA molecules which are dumped into the egg as maternal supply [11].

As far as the MT network in the oocyte is concerned, several conflicting models were published how MTs are organized and how they transport maternal factors to the anterior or posterior ends of the oocyte [12]–[17]. More recently, however, using *in vivo* imaging, Parton et al. resolved many issues of the conflicting models and reported highly dynamic MTs that are organized with a biased random polarity that increased toward the posterior [18]. However, it is important to note that at the end of oogenesis, all MTs are completely degraded again [19]–[21], implying that fertilized embryos need to start to build up the MTs from the beginning.

The ARTS model postulated a mechanism based on MTs that transport the *bcd* mRNA [3]. Yet, so far convincing evidence for such a postulated network of MTs has been lacking. A plethora of information on the content and appearance of *Drosophila* Tubulin is available, but most reports are devoted to the blastoderm stage, while data on MT architecture in early nc embryos are scarce. [22], [23] reported the existence of an extensive cortical network throughout the embryo. However, there is a major problem with these reports, as Taxol was used to stabilize the MTs which gives an altered picture of the native MT architecture. Hence our quest for a technique that would allow to preserve the native appearance of cortical MTs of early nc embryos.

Using a modified permeabilization and fixation method to rapidly fix and preserve cortical MT structures, we detected MT activity in early nc embryos and demonstrate that (i) there is an extensive MT network exclusively in the anterior half, (ii) this MT network is only formed at the cortex, and (iii) Taxol induces artefacts giving the impression that the egg contains a dense and ubiquitous network of cortical MTs. Further, we show that *aTubulin67C* and the kinesin-like minus-end motor *ncd* are critically important for transport of the *bcd* mRNA in the oocyte and along the embryonic cortex to establish the mRNA gradient.

## Results

### The *bicoid* mRNA gradient

**Figure 1:**
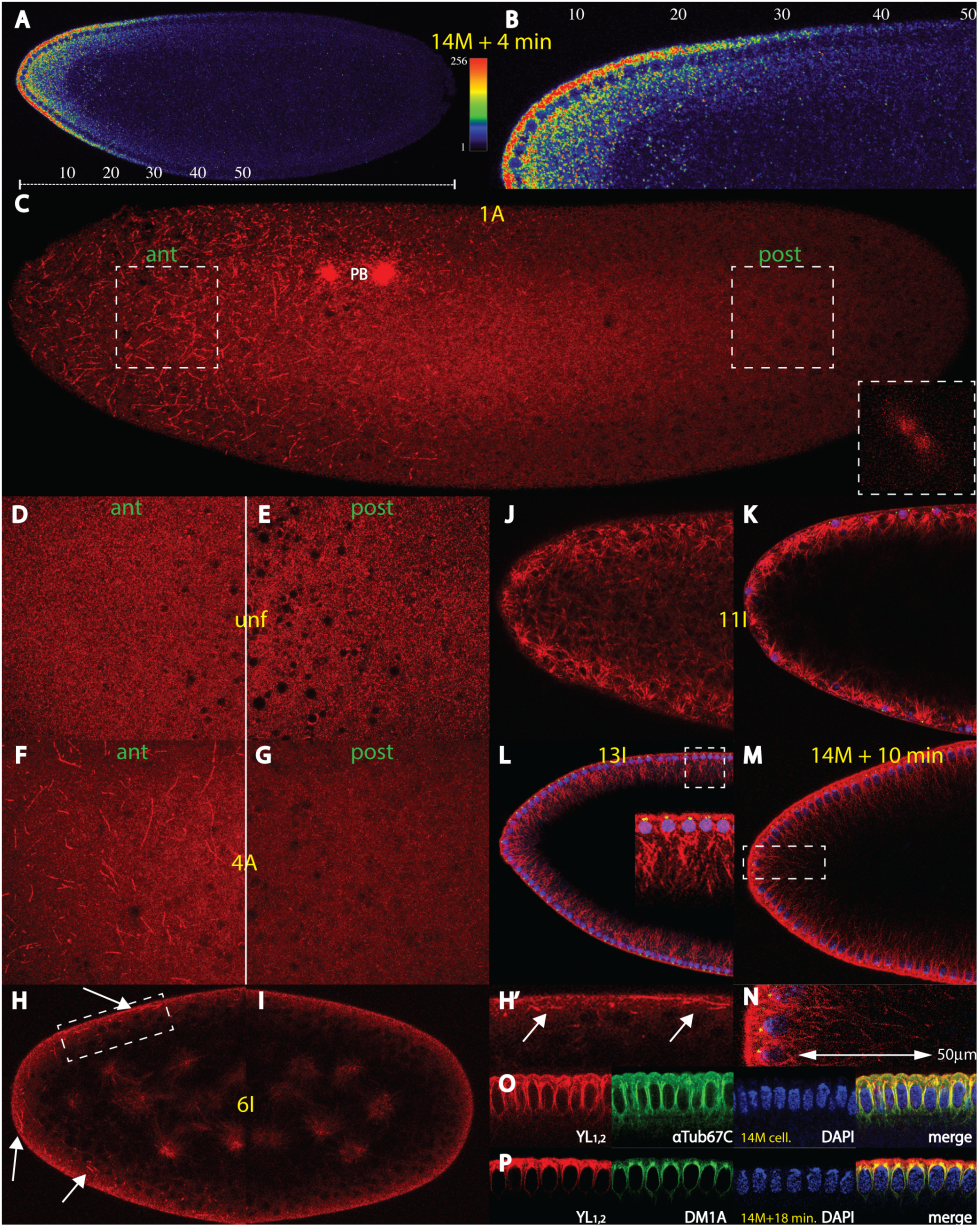
The *bicoid* mRNA gradient and an anterior cortical microtubular network. (A) A single confocal section of a nuclear cycle (nc) 14+4 min. *Drosophila* embryo showing a typical *bcd* mRNA gradient extending up to 50% of egg length (EL, scale bar below embryo). Fluorescence intensities, reflecting *bcd* mRNA concentrations were converted to a colour scale shown at the right (cf. Material and Methods). (B) High magnification of the dorsal region of embryo in (A), numbers of EL above the embryo. (C) Confocal analysis at the surface of a nc 1 embryo at anaphase (insert on lower right) using mab YL_1,2_ against tyrosinated microtubules (MT), showing a network exclusively in the anterior half of the embryo, along with the polar bodies (PB). Two adjacent confocal sections 2.48 µm apart and maximal intensity projection were used. White areas denote magnifications of corresponding anterior (ant) regions and posterior (post) regions used in (D–G). (D) and (E) magnification of anterior (D) and posterior (E) portions on the surface of an unfertilized embryo. No network is visible. (F) and (G) magnification of anterior (F) and posterior (G) portions on the surface of an embryo at nc 4. The network is visible at the anterior half, but is absent in the posterior half. (H), (H’) and (I) sagittal confocal sections of the anterior (H) and the posterior (I) half of an embryo at nc 6. (H’) is a magnification of the area indicated in (H). The MT threads are exclusively at the anterior cortex (arrows). In the interior, asters of interphase nuclei are seen. (J) confocal section just below the nuclear layer of a nc 11 embryo and (K) mid-sagittal section of the same embryo, supplemented with the signal from the nuclear stain using DAPI (blue) and an antibody against γTubulin (green). A dense network is detected around the nuclei. (L) confocal mid-sagittal section of a nc 13 embryo, along with the signals from the nuclear stain using DAPI (blue) and an antibody against γTubulin (green). An extensive MT network ranging deeply into the yolk is visible (insert, thickness about 30 µm). (M) and (N) confocal mid-sagittal section of a nc 14+10 min. embryo, along with the signals from the nuclear stain DAPI (blue) and an antibody against Minispindles (green). (N) high magnification of the insert in (M) showing an extensive MT network ranging deeply into the yolk (insert, about 50 µm deep). (O) colocalization of mab YL_1,2_ to polyclonal αTub67C antibody in a cellularised nc 14 embryo, along with DAPI staining. Most YL_1,2_-positive threads are also positive for αTub67C. (P) colocalization of mabYL_1,2_ to mab DM1A in a nc 14+18 min. embryo, along with DAPI staining. Most YL_1,2_ threads are co-localized with DM1A at perinuclear MTs, while the periplasm is virtually free from αTub84B & D. Stages of embryos are denoted in yellow and follow the nomenclature of [61] and [3].

We recently reported the existence of a *bcd* mRNA gradient in *Drosophila* [3] that corroborated earlier data [2]. Typical examples of an mRNA gradient are shown in an embryo at nc 14+4 min. (Fig. 1A, B, Fig. S1B) where the posterior extent of the gradient reaches about 40% egg length (EL). At this stage, the majority of the mRNA is transported from the basal to apical side of the nuclei and thus accumulates at the periplasm, before rapid degradation commences [3]. At fertilization, however, the mRNA is tightly associated with the tip of the embryo (Fig. S1A). Hence, to convert the pattern at fertilization to that at nc 14, a transportation system was proposed involving cortical MTs which is one of the facets of the ARTS model [3]. In the large blowfly *Lucilia sericata*, the gradient appears very similar (Fig. S1C, D), suggesting that the mRNA gradient as well as the ARTS model are universal among Diptera. Moreover, recent large-scale analysis of cryo-sliced embryos confirms the existence of a long *bcd* mRNA gradient showing transcripts up to 40% EL [24]; FlyBase).

### A cortical MT network in the anterior half of early nuclear cycle embryos

In search for a method to stain native MTs without the necessity to use Taxol for stabilization, we utilized an efficient permeabilization protocol that avoids heptane [25] and adapted it using high concentrations of formaldehyde to “freeze” the cortical MTs as fast as possible. This fixation technique, along with monoclonal antibody YL_1,2_ against tyrosinated tubulin (i. e. against freshly-assembled Tubulin [26], allowed for the staining of a cortical and omnidirectional MT network (Fig. 1C, Video S1). The network can also be visualized by using a more conventional 27% formaldehyde/heptane fixation protocol, but with a somewhat poorer preservation of the MTs. Interestingly, this network was confined exclusively to the anterior half of the embryo and appeared only during metaphase and anaphase of nc 1–6 embryos (Fig. 1C, insert), i. e. during short nuclear phases of 1 minute each [27]. Moreover, the threads were found in the outermost 20 µm of the cortex only (Fig. 1H’). Attempts to live-image the network using αTubulin84B-GFP embryos failed, due to the weakness of the signal and the dynamics of the MTs (data not shown). The network was not detected in unfertilized embryos (Fig. 1D, E), but became apparent from nc 1 onwards, exemplified by a nc 1 (Fig. 1C) or a nc 4 embryo (Fig. 1F). From nc 6 on, it was present throughout all nuclear phases, e. g. during interphase (Fig. 1H, H’), while still being absent in the posterior half (Fig. 1I). At nc 7, when cortical migration commences, the posterior half showed cortical MT activity also (data not shown). At nc 11, tyrosinated tubulin is detected as a dense network below the nuclear layer originating from long astral MTs that surround the nuclei (Fig. 1J, K). Notably, these astral MTs showed Staufen-mediated *bcd* mRNA binding activity [28]. At nc 13, strong MT activity was detected showing long extensions up to 30 µm into the yolk (Fig. 1L, insert) and even longer ones in nc 14 embryos (Fig. 1M, N, up to 50 µm). These threads resemble those on the drawings by [29]. The majority of these long MTs could also be stained using antibodies against αTubulin 67C (αTub67C; Fig. 1O), a maternal Tubulin known to contribute to long MTs and which is expressed mainly in oocytes and in early embryos [30], [31]. nc 14 embryos also revealed a striking regionalization of Tubulin; if stained with mab DM1A which detects both αTubulin 84B and αTubulin 84D (Fig. 1P; [31], then these two Tubulins are preferentially associated with MTs that embrace the nuclei (Fig. 1P, green channel), compared to αTub67C which also accumulates in the periplasm (Fig. 1O, green channel). Hence, the composition of αTubulins in the different cellular sub-compartments varies in space. Since the lengths of these threads by far exceed the observed lengths of MTs of previous reports [22] despite Taxol being used in these studies which favours long MTs, we concluded that the long MTs in Fig. 1J–M were preserved owing to the improved fixation method.

### Independence of the MT network from actin

**Figure 2:**
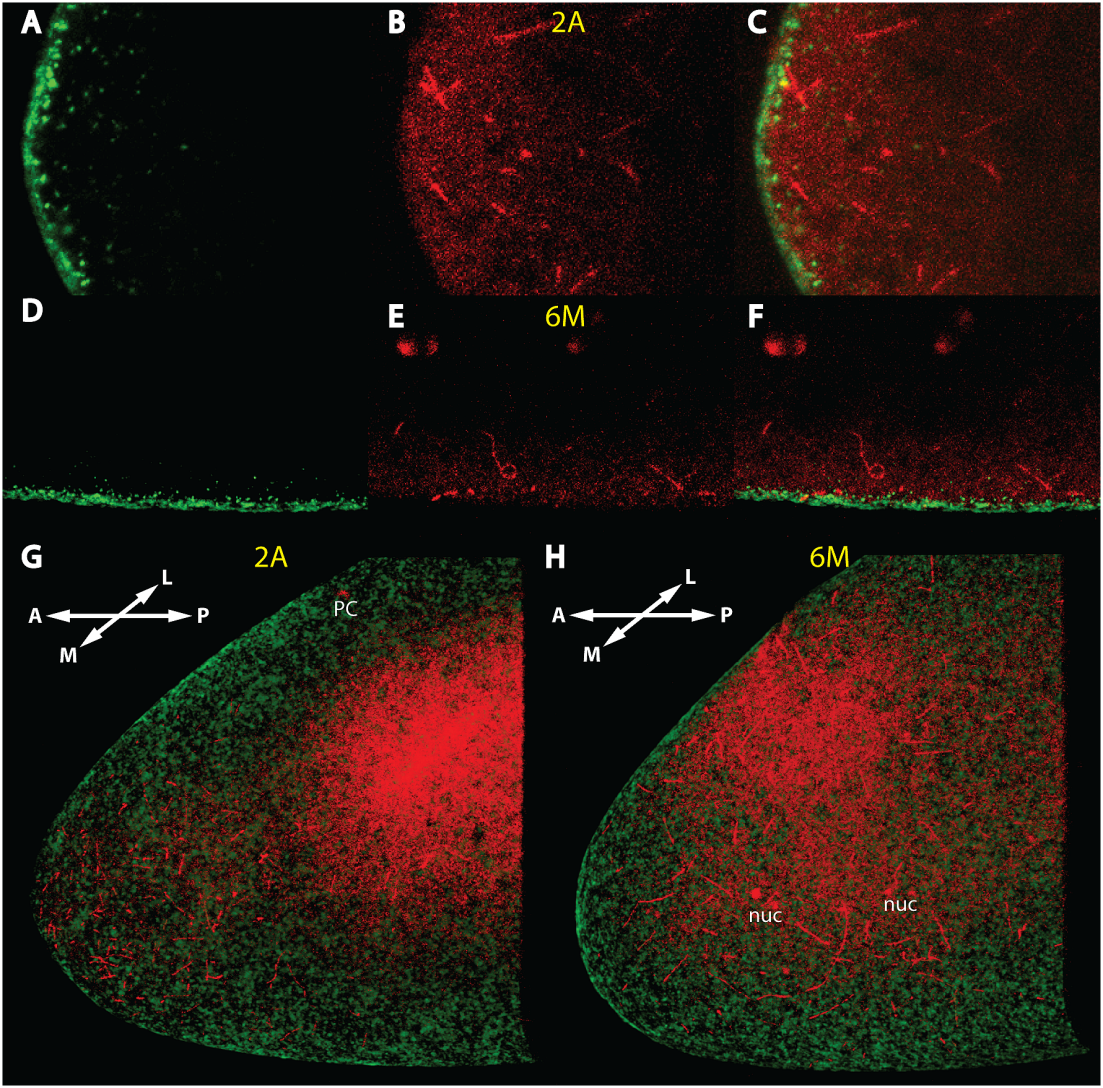
Independence of the early MT network from the actin sheet. (A)–(C) mid-sagittal confocal sections of the anterior tip of a nc 2 embryo stained with Phalloidin (A) to reveal the actin structure, with mab YL_1,2_ against tyrosinated Tubulin (B) and merge in (C). (D)–(F) mid-sagittal section of a ventral region about 50 µm away from the anterior tip of a nc 6 embryo, stained with Phalloidin (D), mab YL_1,2_ (E) and merge in (F). (G) 3-D reconstruction of the confocal stack of the embryo of (A)–(C), view is from the middle (M) to the more lateral (L) part of the embryo. For film of 3D view, see Video S2. (H) 3-D reconstruction of the confocal stack of the embryo of (D)–(F), view is identical as in G. For film of 3D view, see Video S3. The red background on the inner “roof” of the embryos in (G) and (H) is excess of free tubulin which could not be removed during background subtraction of the 3D-program. Stages of embryos are denoted in yellow and follow the nomenclature of [61].

To determine whether or not a link exists between the cortical MT network and actin, we examined their relative distribution patterns in early embryos. In the anterior tip of a nc 2 embryo, the actin formed a dense layer (Fig. 2A) which was not in contact with the MT network (Fig. 2B, C). At later stages (nc 6), the situation remained unchanged (Fig. 2D–F). A 3-D visualization technique of the two confocal stacks in Fig. 2A and B allowed us to visualize the relationship of the MT threads to actin more explicitly (Fig. 2G, H; Video S2 & S3). Evidently, at no location of the scanned area, the MT network was associated with the actin sheet, rather it resided immediately next to it at the inner cortex.

### Taxol induces artefacts

**Figure 3:**
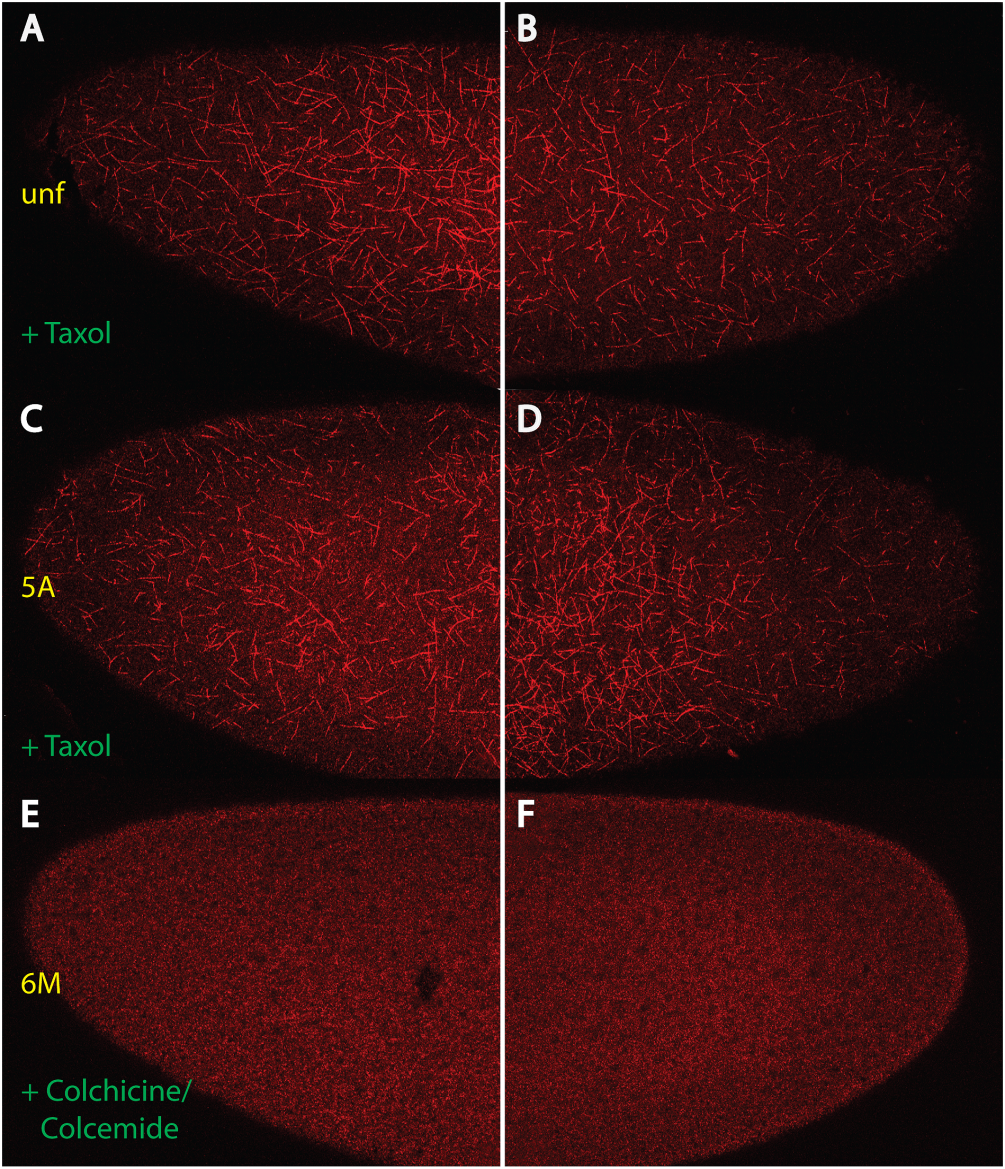
Behaviour of the MT threads upon treatment with drugs: use of Taxol results in artefacts. Anterior (A, C, E) and posterior (B, D, F) ends of embryos treated with Taxol (A–D) or Colchicine & Colcemide (E, F) stained with mab YL_1,2_ to reveal the MT network. (A, B) unfertilized embryo, (C, D) nc 5 embryo, (E, F) nc 6 embryo. Note that Taxol induces the formation of threads even in unfertilized embryos (compare to Fig. Fig. 1D, E) and even in posterior halves (B, D). Stages of embryos are denoted in yellow and follow the nomenclature of [61].

Previous analyses of MT activity reported a cortical network of short MT [22], [23], [32], [33], but many of these investigations used the MT-stabilizing drug Taxol. We therefore repeated these experiments with Taxol and noted that the MT threads could even be detected in unfertilized embryos (Fig. 3A, B). Furthermore, the threads were found uniformly distributed in the posterior half. Fertilized embryos showed the same distribution, as shown in a nc 5 embryo, although the density of threads was somewhat increased, while no difference in length was detected (Fig. 3C, D). When a mixture of Colchicine and Colcemide, drugs known to destabilize MT threads, was applied, we found that the drug treatment led to complete degradation of all MT threads (Fig. 3E, F). Our data from Taxol treatments suggest that the Tubulin monomers are present throughout the embryo, but under normal conditions they polymerize only in the anterior half.

### *αTubulin67C* and *ncd* are critically important for cortical MT formation and *bcd* mRNA transport

**Figure 4:**
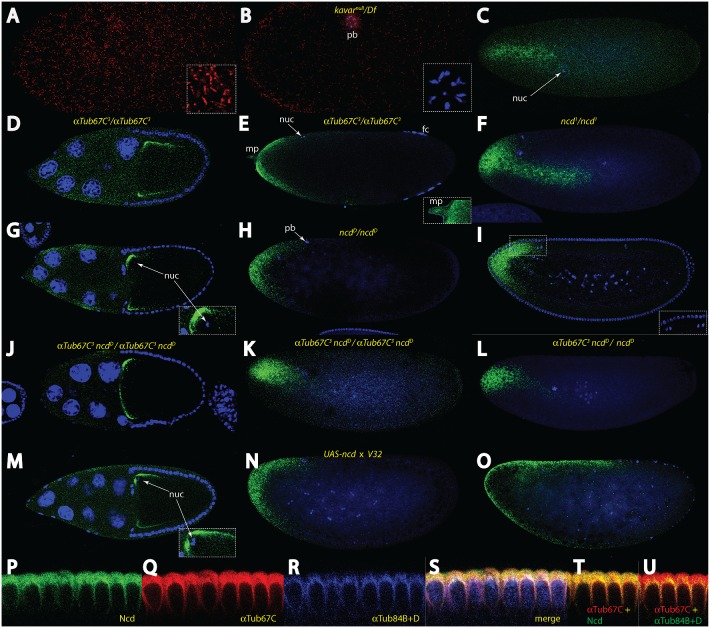
*α tubulin 67C* and *ncd* are essential for transport of the *bcd* mRNA. (A) surface confocal section of an anterior half of a *kavar^null^*/*Df(3L)55* embryo, stained with mab YL_1,2_ revealing a short omnidirectional MT network (insert). (B) more sagittal confocal section of the same embryo as in (A) to reveal the polar body (pb), stained along with DAPI to show the chromosomes (insert). Note the short MT threads at the cortex. (C) a *kavar^null^*/*Df(3L)55* embryo, stained for the *bcd* mRNA (green) along with DAPI (blue) to reveal the nucleus (nuc). (D) stage 10 and (E) stage 14 *αTub67C^3^/αTub67C^3^* oocytes stained for *bcd* mRNA (green) and DAPI (blue). Insert in (E) denotes staining in the micropyle (mp). (F) nc 4 *ncd^1^*/*ncd^1^* embryo, stained for the *bcd* mRNA (green) along with DAPI (blue). (G–I) *ncd^D^/ncd^D^* stage 10 oocytes (G), *ncd^D^/ncd^D^* nc 6 embryo (H) and *ncd^D^/ncd^D^* nc 13 embryo (I), stained for *bcd* mRNA (green) and DAPI (blue). Insert in (G) denotes the position of the nucleus on the dorsal side. Insert in (I) denotes irregularities in the position of nuclei frequently seen in *ncd^D^/ncd^D^* embryos. (J–K) homozygous *αTub67C^3^ ncd^D^* stage 10 oocyte (J) and nc 4 embryo (K) stained for *bcd* mRNA (green) along with DAPI (blue). (L) *αTub67C^3^ ncd^D^*/*ncd^D^* nc 6 embryo stained for *bcd* mRNA (green) along with DAPI (blue). (M–O) *UAS-ncd* overexpression using maternal GAL-4 driver *V32*, stained for *bcd* mRNA (green) and DAPI (blue), stage 10 oocyte (M), nc 6 embryo (N) and nc 12 embryo (O). Insert in (M) denotes the position of the nucleus on the dorsal side. (P–U) mid-sagittal section of a nc14 embryos stained for Ncd (P), αTub67C (Q), αTub84B+D (R), and merge of P-Q, along with DAPI (S). Note that Ncd, αTub67C and αTub84B+D overlap only in MTs surrounding the nuclei (white). (T) Ncd staining together with αTub67C. (U) αTub67C staining together with αTub84B+D. Note that Ncd overlaps with αTub67C (T), in contrast to αTub84B+D (U). fc follicle cells, nuc nuclei, mp micropyle, pb polar body.

Of the *αTubulin* genes [34] that would be critical for cortical mRNA transport, we considered *αTub67C* a good candidate since it showed maternal expression and an expression profile similar to that of *bcd* mRNA [34], [35]. The *αTub67C* locus is represented by the *αTub 67C* mutant alleles [30], [36] and by the independently-isolated dominant female-sterile *Kavar* mutants [31], [37]. We stained eggs of *kavar^null^/*- hemizygous mothers (a complete loss-of-function (LOF) mutation) [37] for the presence of the cortical network and found a network of short MTs without directionality (Fig. 4A, B), consistent with the notion that *αTub67C* is required for the formation of long MTs [31], [37]. If assayed for *bcd* mRNA transport, embryos do not show any cortical transport, rather the mRNA formed a long streak in the interior of the embryo in parallel to the A-P axis (Fig. 4C). Next, we assayed the behaviour of the mRNA in oocytes of homozygous *αTub67C^3^* females, the weakest available allele. In a stage 10 *αTub67C^3^* oocyte, the mRNA did not localize to the anterior pole, residing instead at the edge (Fig. 4D). In a late stage 14 oocyte, the aberrant lateral localization was even more pronounced showing a shallow anterior distribution (Fig. 4E), demonstrating a vital role for *αTub67C^3^* allele in anterior localization of the *bcd* mRNA during oogenesis, in contrast to the *null* alleles, see below. In *αTub67C^3^* embryos, the mRNA shows a shallow gradient from the time of fertilization on, similar to the profile in late oogenesis (Fig. 4E, data not shown). However, when *αTub67C^3^* embryos were assayed for the presence of the cortical network using mab YL_1,2_, we noted a dense network of MTs (Fig. S2C). A larger area showed the MTs assembled in aster-like structures (asteriks), surprisingly without any nuclei in their centers, previously detected in *αTub67C^2^* and *αTub67C^4^* alleles [30]. The remaining area showed a dense network, similar to Taxol-treated embryos (Fig. 3A–D). The other *αTub67C* alleles behaved similar to the LOF allele, with *αTub67C^1^*, *αTub67C^2^*, *Kavar^21G^* and *Kavar^18C^* showing the mRNA streak (Fig. S2A, B D, E). Interestingly, *αTub67C^3^* was the only allele that showed a specific effect on mRNA transport and localization in the oocyte (Fig. 4D, E), while the *null* allele showed the mRNA accumulated largely normal at the anterior pole during oogenesis (Fig. S2H). This notion is supported by data from [38] that demonstrated that *kavar^nul^* ^l^ mutants show almost normal ooplasmic streaming.

In search for specific motor proteins associated with αTub67C, we noted the *nonclaret disjunctional* (*ncd*) locus encoding an unconventional Kinesin which transports cargos to the minus-end [39] and which interacts genetically with *αTub67C* [40], [41]. We first analysed a strong *ncd* allele, *ncd^1^* which again showed a large *bcd* mRNA streak (Fig. 4F), similar to that in *kavar^null^* mutant embryos (Fig. 4C). A semi-lethal allele, *ncd^05884^*, showed this streak less pronounced and some cortical movement was observed (Fig. S2F). Next, we analysed oocytes from *ncd^D^/ncd^D^* homozygous females, a weak semi-dominant female sterile allele [42] and noted that in stage 10 oocytes, the *bcd* mRNA accumulated laterally (Fig. 4G), although not as severe as in *αTub67C^3^* oocytes (Fig. 4D). Consequently, due to the lateral localization in oocytes, in early nc 6 *ncd^D^/ncd^D^* embryos, the *bcd* mRNA appeared more posterior (Fig. 4H), compared to wild-type embryos of the same stage, while in nc 13 embryos, this aberrant localization appeared largely corrected to wild-type distribution (Fig. 4I). In oocytes of homozygous *αTub67C^3^ ncd^D^* mothers, anterior mRNA transport in oocytes appeared rather normal (Fig. 4J), suggesting that *ncd^D^* ameliorates the *αTub67C^3^* phenotype. However, when embryos derived from homozygous *αTub67C^3^ ncd^D^* mothers were assayed, they again revealed complete absence of cortical *bcd* mRNA transport (Fig. 4K). Rather, the mRNA followed a path towards the aberrantly-looking nuclei in the center of the yolk, reminiscent to the situation in *kavar^null^* or *ncd^1^* embryos (Fig. 4C, F), however less pronounced. In *αTub67C^3^/+ncd^D^*/*ncd^D^* embryos [41], the mRNA followed a similar path to the center (Fig. 4L), consistent with the dominant phenotype of *αTub67C* mutations [30], [37].

To further corroborate an involvement of *ncd* in the transport of *bcd* mRNA, we over-expressed Ncd during oogenesis using the maternal driver V32. A stage 10 oocyte showed lateral accumulation of the mRNA (Fig. 4M), similar to *αTub67C^3^* oocytes (Fig. 4D) and to a lesser extent also to *ncd^D^* oocytes (Fig. 4G). As a result of the aberrant lateral transport during oogenesis, a nc 6 embryo revealed the mRNA transported more posteriorly (Fig. 4N) compared to a wild-type embryo of the same stage, and even more posterior than an identical stage *ncd^D^*/*ncd^D^* embryo (Fig. 4H). In a nc 12 embryo, however, extended transport of the mRNA at the cortex well beyond the middle of the embryo was observed (Fig. 4O), demonstrating a vital role of *ncd* for the transport of the mRNA. We also noted a clear bias for accumulation of the *bcd* mRNA at the dorsal side in oocytes, exemplified by the position of the oocyte nucleus (Fig. 4G, M, inserts) which explains the skew of the *bcd* mRNA pool at the time of fertilization (Fig. 1B, insert).

### αTubulin67C and Ncd show colocalization

To perfect our analysis on the proposed molecular interaction of *αTub67C* with *ncd* [40], [41], we stained embryos with antibodies specific for each protein alone, along with a third antibody that recognized αTub84B and αTub84D (Fig. 4P–U). Ncd and αTub67C showed colocalization in the periplasm and in perinuclear areas of nc 14 embryos (Fig. 4P, Q). Notably, the periplasm also showed strong *bcd* mRNA accumulation upon basal-apical transport (Fig. 1B; [3]), suggesting that αTub67C and Ncd play also a role during basal-apical transport at nc 14. In contrast, αTub84B and D localized immediately perinuclear, but were virtually absent from the periplasm, as evident in Fig. 4R. Consequently, only the perinuclear area was positive for all 3 proteins which stained in white (Fig. 4S). A pair-wise comparison of Ncd/αTub67C (Fig. 4T) and Ncd/αTub84B+D (Fig. 4U) corroborated this behaviour clearly. The above data suggested that Ncd preferentially associated with αTub67C-positive MT bundles and define these two proteins as essential members of the *bcd* mRNA transport machinery.

## Discussion

The ARTS model [3] predicted the presence of a cortical MT network in early staged *Drosophila* embryos where only sparse information of MT distributions was available. To visualize this network, two essential modifications of existing protocols were necessary: 1) a permeabilization and fixation protocol permitted to overcome the limitations of rapid fixation of MT structures in the *Drosophila* egg, and 2) an antibody with an excellent signal-to-noise ratio that detected tyrosinated Tubulin.

Initially, visibility of the cortical network was limited to a short 2 minute-window which let us conclude that it is a highly dynamic and short-lived MT network, consistent with the reported specificity of the mab against tyrosinated Tubulin [26]. The rapid fixation technique also disclosed MT activity with MT extensions as long as 50 µm into the yolk at later stages (Fig. 1M, N). The MT threads likely serve to transport *bcd* mRNA molecules from the yolk to the apical side of the nuclei at early nc 14 [3]. Another possible function could be transporting lipid droplets, shown to be MT-based [43], [44].

Freshly-synthesized Tubulin usually carries a COOH-terminal tyrosine residue as signature which is part of the epitope that is recognized by mab YL_1,2_. Mature tubulin shows this terminal tyrosine cleaved off, often referred to as Glu-Tubulin, using an enzyme, Tubulin tyrosine carboxypeptidase (TTCP). If necessary, tyrosination of the de-tyrosinated end using an enzyme, Tubulin tyrosine ligase (TTL) could convert the COOH-end back to its original status (reviewed by [45]). As there is no true TTL detected in the *Drosophila* genome, we presume that tyrosinated Tubulin is provided to the egg via the pool of maternally-deposited Tubulin, or *de novo* synthesis.

Our Taxol experiments in Fig. 3 demonstrated that most previous descriptions of MT activity in the early nc embryo [22], [23], [32], [33] require a careful re-examination. The fact that Taxol could induce MT threads even in unfertilized embryos (Fig. 3A, B) suggested that the unfertilized egg was competent for MT thread assembly, but lacked an anteriorly-located trigger, or ‘initiator’ which is activated only after fertilization.

Attempts to counterstain the cortical MT threads with minus-end markers failed (data not shown), most likely because there is no ‘conventional’ microtubule organizing center (MTOC) at the cortex. Notably, the same minus-end markers usually detected the MTOCs associated with the internal nuclei without difficulty. Conversely, antibodies against plus-end markers do not stain well following our improved fixation method. We were also unable to stain the MT threads using Tubulin-GFP-constructs (unpublished), most likely due to the fact that the signal from their fine structures is difficult to detect within the autofluorescence of the yolk. The only protein that co-stains the cortical MT network is Chromosome bows (Chb), formerly called Mast/Orbit/CLASP [46], [47], a MT-binding protein and a plus-end marker, which decorated the MT threads uniformly (data not shown). A recent survey in the literature revealed compelling evidence that MTs can form without functional centrosomes [48]–[53]. Of these, the observation that MTs can be nucleated at the *trans*-Golgi network with the help of vertebrate CLASP [53] attracted our attention. Indeed, Golgi network structures could be detected at the cortex of early nc 1–6 embryos [54], [55].

As far as the velocity of the *bcd* mRNA transport is concerned, the velocity of Staufen-*bcd*-RNP complexes was measured in the oocyte, resulting in values of 0.36 to 2.15 µm/sec. [10]. As was argued in favour of the ARTS model [3], an omnidirectional MT network was predicted as a prerequisite for the transport system, due to the fact that an A-P directed MT network would have transported the *bcd* mRNA within minutes from the anterior to the posterior pole. As was discussed for the ARTS model [3], a slight directional bias of the MT network, as was observed in oocytes during Stau-*oskar* mRNA particle transport [17], represents a valid model for posterior transport, but only if the transport is mediated by both plus- and minus-end motors. However, what the ARTS model could not foresee was the fact that the transportation system would persist only during about 20% of the time of an early nuclear cycle.

An important facet is that all MT activities in the oocyte are abolished at the time of fertilization [19]–[21]. Hence, new MT structures need to be built up in the fertilized egg. Our data demonstrate the existence of at least two distinct and undiscovered MT networks in the early embryo: (i) the cortical network that relies critically on the process of fertilization and *αTub67C* (Figs. 1, 4), (ii) an internal network which rapidly transports the mRNA towards to the interior of the embryos, but not requiring functional αTub67C nor Ncd. Since the latter MT network is located in the middle of the embryo, its detection proves difficult and hence still awaits visualization. Hints for its existence were shown in the initial inward movement of the *bcd* mRNA during nc 3–4, as noted by [3], [56], before the cortical network comes into play which transports the mRNA to the cortex [3], [56]. Interestingly, about 1–1.5% of all fertilized wild-type embryos show aberrant transport of the mRNA into the interior and towards the dividing nuclei (data not shown), suggesting that this network may correspond to that proposed for axial expansion of nuclei at nc 4–6 [57].

Fig. 1A, B corroborated earlier findings of a basal-to-apical transport of the mRNA during nc 14 and the existence of an apically-located *bcd* mRNA [2], [3], before its final degradation takes place. This behaviour was negated in the analysis of [56]. Curiously, embryo G in Fig. S1 of the same report demonstrated strong expression of an apical *bcd* mRNA, thus making the claim of a basal degradation of the mRNA without apical transport inconsistent. How do we reconcile these opposing results? We surmise that the discrepancy between our result demonstrating the presence of an apical mRNA and their result showing absence was caused by the fact that many embryos in the Little et al. analysis revealed poor preservation of the periplasm which appeared largely rubbed off, best exemplified in embryo E and F of their Fig. 5, respectively. Consequently, little or no fluorescent signal could emerge from the missing tissue. As far as the discrepancy of a long versus a short mRNA gradient is concerned, we surmise (i) a similar cause owing to the rubbed periplasm, and (ii) possibly a general loss of mRNA of the poorly-preserved embryos. Indeed, the long mRNA gradient was most conspicuous from the apical mRNA species [3]; Fig. 1A, B). The observed short mRNA gradient led Little et al. to conclude that diffusion of the Bcd protein is still an absolute requirement for fulfilment of the SDD model. On the other hand, our analyses [3]; this report) and that of [24] demonstrated that the mRNA gradient reached a much more posterior extent that is sufficiently large to exclude the necessity to include long-range diffusion of the Bcd protein. To reconcile the difference of the posterior extent between the mRNA and the protein gradient, we propose that the mRNA prepatterns the protein gradient. Furthermore, we envision that the poly(A) tail length of the mRNA and its control of the translation efficiency of Bcd [58] may represent another tool to extend the gradient to the posterior. Thus, we speculate that the length of the poly(A) tail of the *bcd* mRNA increases with posterior migration such that the posterior-most particles harbour the longest poly(A) tail. As a consequence, only few mRNA molecules would be required to efficiently translate the Bcd protein in more posterior regions, molecules that current *in situ* hybridization protocols are unable to detect. Interestingly, Wispy, a poly(A) polymerase was recently shown to be part of the *bcd* ribonucleoprotein particle [59]. Possibly, Wispy remains associated with the particle during the posterior migration, while simultaneously regulating the elongation of the poly(A) tail of the *bcd* mRNA. Like this, the poly(A) tail length of the *bcd* mRNA does not only vary with time [58], but may also vary with space.

To take up the question that was asked in the introduction: we demonstrate that the molecular structures which the ARTS model predicted exist and described two proteins that play critical roles for the transport machinery that form the *bcd* mRNA gradient. Further, we provide evidence of a long-range transport of *bcd* mRNA particles up to 40% EL [3] culminating in the mRNA gradient which largely dictates the protein gradient. Hence, the crucial question of the ARTS model concerning the mechanism by which the *bcd* mRNA gradient forms, is answered and confirms the earlier postulate of a MT-mediated transport [3].

## Materials and Methods

### Fly stocks

*kavar^rX21^*, referred to as *kavar^null^*, the only known null allele of *αTub67C* is described in [37] and was used, together with *Df(3L)55*, to generate embryos without functional *αTub67C* activity [31], [37]. The other *kavar* alleles were described in [37]. The *αTub67C^1^, αTub67C^2^* and *αTub67C^3^* alleles were described in [30], the *ncd^D^*, *ncd^1^* and *αTub67C^3^ ncd^D^* double mutants in [40], [42], respectively. *αTub67C^3^, ncd^D^/ncd^D^* embryos were obtained by crossing *αTub67C^3^ ncd^D^*/+females to *ncd^D^/ncd^D^* males, as described by [41]. The semi-lethal *ncd^05884^* allele and the over-expression P(EPgy2)*ncd^EY03397^* allele were obtained from Bloomington. The maternal GAL4-driver line *V32* was obtained from the Perrimon lab.

### Fixation of embryos to visualize the microtubular network

In order to visualize the cortical network, we modified a recent permeabilization protocol without heptane [25] and fixed embryos for 15 minutes in 27% formaldehyde, followed by gentle devitellinization. The network can also be visualized by using a more conventional 27% formaldehyde/heptane fixation protocol, but with a somewhat poorer preservation of the MTs. Embryos in Fig. 2 were fixed like the other embryos, but were hand-devitellinized.

### Drug treatments

After dechorionation, embryos were subjected to 10 µg/ml Taxol for 2 minutes, or to a 50 µg/ml Colcemide/20 µg/ml Colchicine mixture for 10 minutes in permeabilization buffer [25], before addition of 27% formaldehyde.

### Antibodies

Mab YL_1,2_ against tyrosinated tubulin (Millipore) was used at 1∶2000. Anti-αTub67C rabbit polyclonal antibodies were made against a peptide from amino acids (aa) 35–61 of αTub67C, affinity-purified and used at 1∶400. Anti-αTub84B+D guinea pig polyclonal antibodies were made against the last 15 aa including the tyrosine residue of the common COOH-termini of αTub84B and αTub84D, respectively, and used as crude serum at 1∶300. DM1A was used as a FITC-conjugate (Sigma) at 1∶50 sequentially to mab YL_1,2_ in Fig. 1P. Polyclonal goat antibodies against Ncd (dS-17) were purchased from Santa Cruz Biotechnology and used at 1∶80. Polyclonal rabbit anti γTubulin and Msps antibodies were obtained from Y. Zheng and H. Ohkura, respectively, and were used both at 1∶1000. For actin staining, we hand-devitellinized the embryos and used Phalloidin, coupled to Alexa 488 (Invitrogen) at 1∶60. For YL_1,2_, we preferentially used 2^nd^ antibodies coupled to 594 nm fluorochromes to obtain an optimal signal-to-noise ratio. All pictures were recorded on a Zeiss LSM 710.

### Western analysis

0–2 h extracts were separated on 12% PAGE and probed with Tubulin-specific mab’s and a polyclonal antiserum using standard methods, as described in Figure S3.

### *In situ* hybridization

Fluorescent *in situ* hybridization was used according to [3], except that RNA probes were used, combined with an Alexa Fluor 568 Signal-Amplification Kit (Invitrogen A11066). Care was taken to ensure preservation of the periplasm by gentle vortexing during the devitellinization step and proper fixation during the prehybridization steps to avoid poor preservation of the periplasm as in [56].

### Colour conversion and 3D-analysis

For colour conversion and interpretation of signal intensities in Fig. 1A, B, the OsiriX DICOM program was used [60]. For 3D-analyses in Videos S2–S4, the ZEN 2009 program (Zeiss GmbH) was used. Note that due the coverslip, the surface of the embryos does not appear not round, but rather flat.

## Supporting Information

Figure S1 ***bcd*** **mRNA gradients in Diptera.** (A) a *Drosophila* embryo at fertilization hybridised with a *bcd* probe and alkaline phosphatase to reveal the strict accumulation of the mRNA at the anterior pole. (B) a *Drosophila* nc 14 embryo hybridised with a *bcd* probe and alkaline phosphatase showing an extended gradient. (C) a *Lucilia sericata* nc 14 embryo hybridised with a *bcd* probe and alkaline phosphatase showing an extended gradient. (D) a *Lucilia sericata* nc 14 embryo hybridised with a *bcd* probe using fluorescence. Methods and colour conversion as in Fig. 1A, B and [3].
(TIF)

Figure S2 **Mislocalization of the** ***bcd*** **mRNA in** ***αTub67C*** **and motor protein mutants.** (A) a nc 1 *αTub67C^1^/αTub67C^1^* embryo, stained for the *bcd* mRNA (green) along with DAPI (blue) to reveal a streak of the mRNA. (B) a nc 5 *αTub67^2^/αTub67C^2^* embryo, stained for the *bcd* mRNA (green), along with DAPI (blue). (C) 3-D reconstruction of the tip of a nc 5 *αTub67C^3^/αTub67C^3^*embryo, stained with mab YL_1,2_ (red) and DAPI (blue) to reveal a dense MT network and aster-like MT bundles without nuclei (asteriks). The positions of the nuclei are indicated with yellow arrows, one normal metaphase nucleus is indicated with a white arrow. A movie of this 3-D construction is available as Video S4. (D) a nc 5 *Kavar^21G^*/*+* embryo, stained for the *bcd* mRNA (green), along with DAPI (blue). (E) a nc 1 *Kavar^18C^*/*+* embryo, stained for the *bcd* mRNA (green), along with DAPI (blue). (F) a nc 3 *ncd^05884^*/*ncd^05884^* embryo, stained for the *bcd* mRNA (green) along with DAPI (blue). (G) wild-type stage 10 oocyte, stained for the *bcd* mRNA (green), along with DAPI (blue). (H) *kavar^null^*/*Df(3L)55* stage 10 oocyte, stained for the *bcd* mRNA (green), along with DAPI (blue). The anterior localization is largely normal.
(TIF)

Figure S3 **Specificity of Tubulin antibodies.** Western analysis of 0–2 h embryonic extracts (Fig. S3) showed that mab YL_1,2_ detected two Tubulin bands, in accordance with previous reports [62]. The upper band corresponded to αTub67C, while the lower band corresponded to both αTub84B and αTub84D [31]. mab DM1A, another αTubulin-specific mab specifically detected the lower αTub84B/D band, in accordance with [31], while the αTub67C-specific-antibody detected exclusively the upper band.
(TIF)

Video S1 **An exclusive anterior MT network.** Video of the 3D-reconstruction of the confocal stack used in Fig. 1C to reveal the MT network exclusively in the anterior half of a nc 1 embryo.
(ZIP)

Video S2 **Independence of the early MT network from the actin sheet in a nc 2 embryo.** Video of the 3D-reconstruction of the confocal stack used in Fig. 2A–C, G. The MT network (red) is not in contact with the actin sheet (green) of a nc 2 embryo.
(ZIP)

Video S3 **Independence of the early MT network from the actin sheet in a nc 6 embryo.** Video of the 3D-reconstruction of the confocal stack used in Fig. 2D–F, H. The MT network (red) is not in contact with the actin sheet (green) of a nc 6 embryo.
(ZIP)

Video S4 **Asters and intensive MT network activity at the cortex of** ***αTub67C^3^*** **embryos.** Video of the 3D-reconstruction of the confocal stack used in Fig. S2C. An intense MT activity with huge asters and a dense network is observed (red). Note that the internal nuclei (blue) are not associated with the asters.
(ZIP)
