# Supplementary figures and images for: αTubulin 67C and Ncd Are Essential for Establishing a Cortical Microtubular Network and Formation of the Bicoid mRNA Gradient in Drosophila
Source: PLoS One. 2014 Nov 12;9(11):e112053. doi: 10.1371/journal.pone.0112053 (PMC4229129; doi:10.1371/journal.pone.0112053)

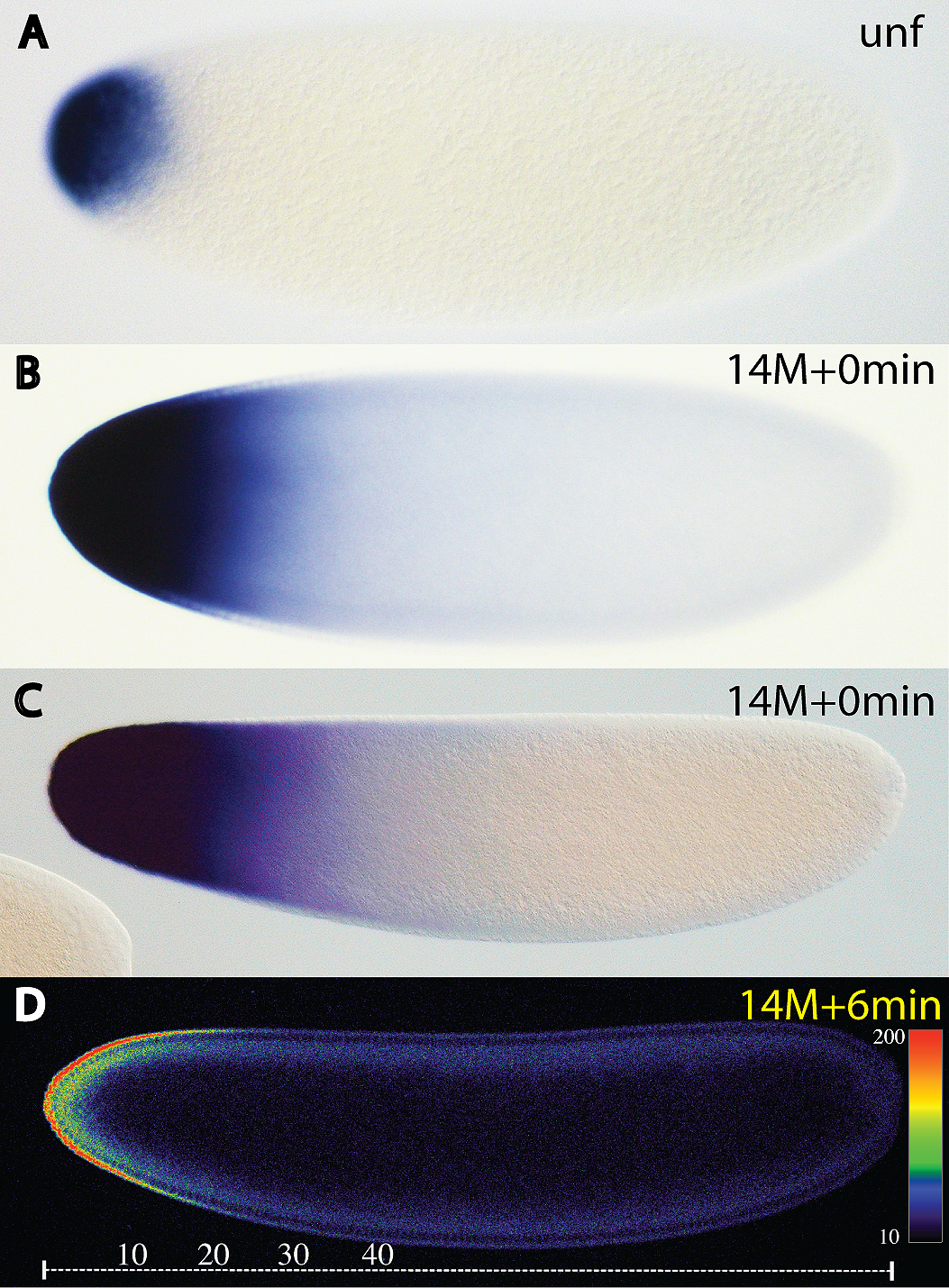

Supplement: Figure S1 — bcd mRNA gradients in Diptera. (A) a Drosophila embryo at fertilization hybridised with a bcd probe and alkaline phosphatase to reveal the strict accumulation of the mRNA at the anterior pole. (B) a Drosophila nc 14 embryo hybridised with a bcd probe and alkaline phosphatase showing an extended gradient. (C) a Lucilia sericata nc 14 embryo hybridised with a bcd probe and alkaline phosphatase showing an extended gradient. (D) a Lucilia sericata nc 14 embryo hybridised with a bcd probe using fluorescence. Methods and colour conversion as in Fig. 1A, B and [3]. (TIF) [file pone.0112053.s001.tif]

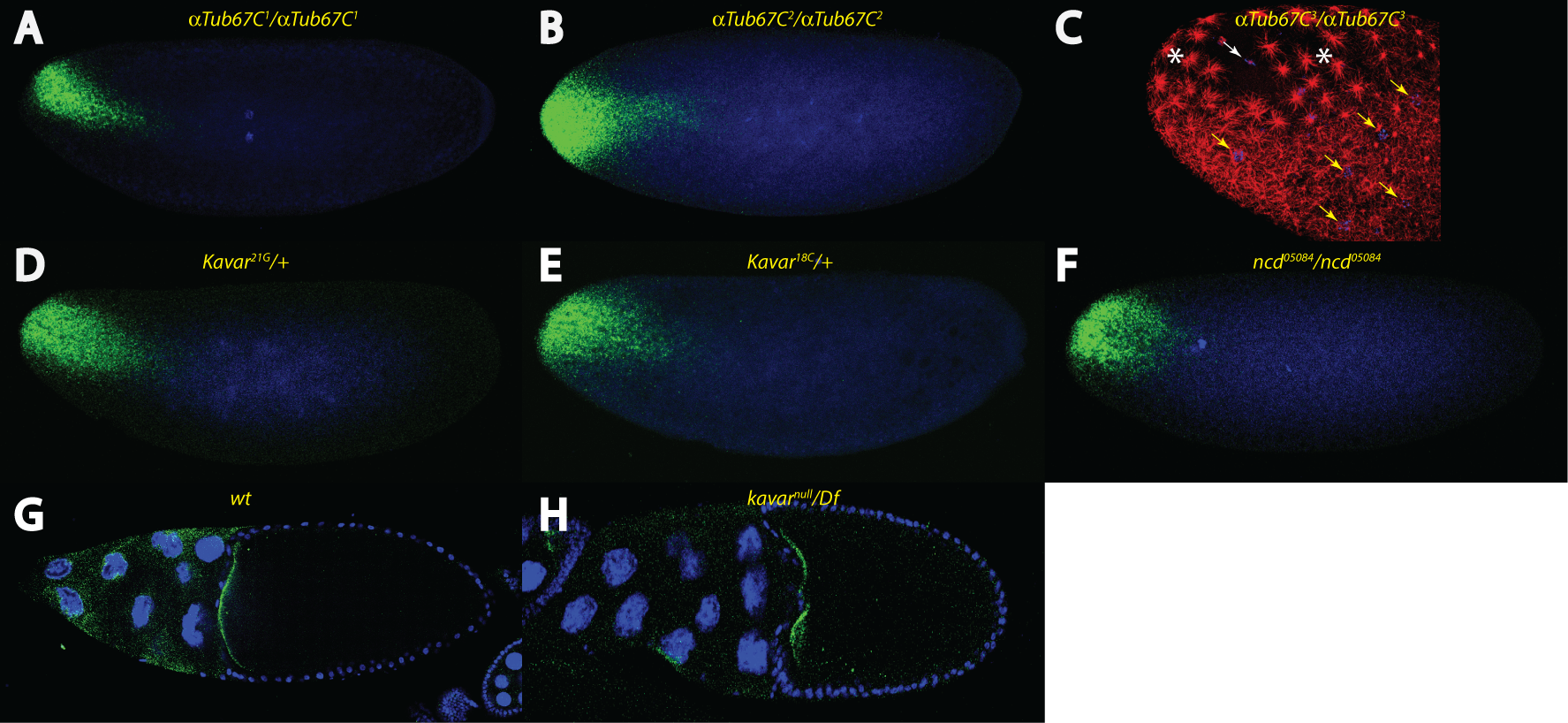

Supplement: Figure S2 — Mislocalization of the bcd mRNA in αTub67C and motor protein mutants. (A) a nc 1 αTub67C1/αTub67C1 embryo, stained for the bcd mRNA (green) along with DAPI (blue) to reveal a streak of the mRNA. (B) a nc 5 αTub672/αTub67C2 embryo, stained for the bcd mRNA (green), along with DAPI (blue). (C) 3-D reconstruction of the tip of a nc 5 αTub67C3/αTub67C3embryo, stained with mab YL1,2 (red) and DAPI (blue) to reveal a dense MT network and aster-like MT bundles without nuclei (asteriks). The positions of the nuclei are indicated with yellow arrows, one normal metaphase nucleus is indicated with a white arrow. A movie of this 3-D construction is available as Video S4. (D) a nc 5 Kavar21G/+ embryo, stained for the bcd mRNA (green), along with DAPI (blue). (E) a nc 1 Kavar18C/+ embryo, stained for the bcd mRNA (green), along with DAPI (blue). (F) a nc 3 ncd05884/ncd05884 embryo, stained for the bcd mRNA (green) along with DAPI (blue). (G) wild-type stage 10 oocyte, stained for the bcd mRNA (green), along with DAPI (blue). (H) kavarnull/Df(3L)55 stage 10 oocyte, stained for the bcd mRNA (green), along with DAPI (blue). The anterior localization is largely normal. (TIF) [file pone.0112053.s002.tif]

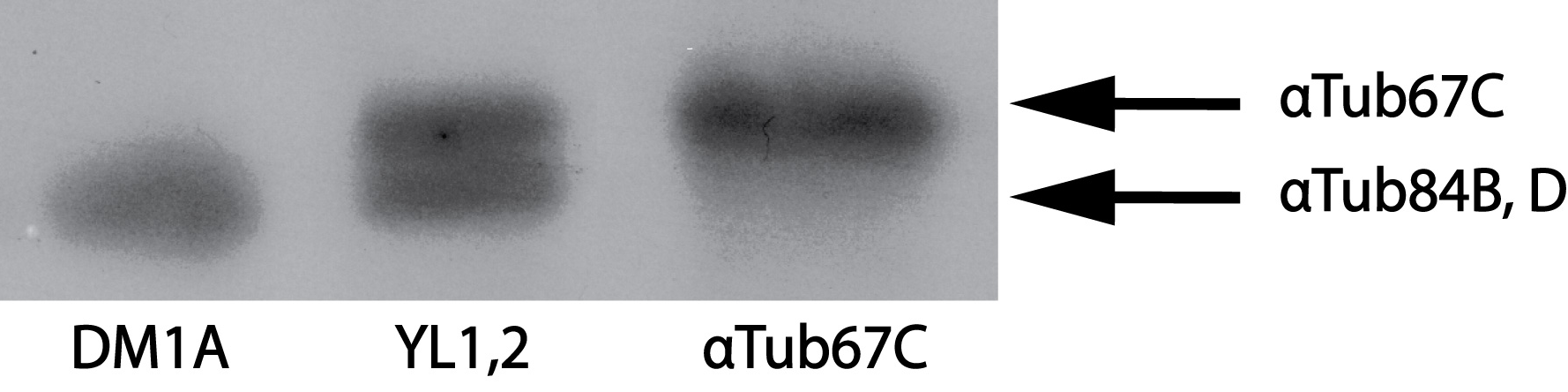

Supplement: Figure S3 — Specificity of Tubulin antibodies. Western analysis of 0–2 h embryonic extracts (Fig. S3) showed that mab YL1,2 detected two Tubulin bands, in accordance with previous reports [62]. The upper band corresponded to αTub67C, while the lower band corresponded to both αTub84B and αTub84D [31]. mab DM1A, another αTubulin-specific mab specifically detected the lower αTub84B/D band, in accordance with [31], while the αTub67C-specific-antibody detected exclusively the upper band. (TIF) [file pone.0112053.s003.tif]
